# Lyophilized human cells stored at room temperature preserve multiple RNA species at excellent quality for RNA sequencing

**DOI:** 10.18632/oncotarget.25764

**Published:** 2018-07-31

**Authors:** Lilla Ozgyin, Attila Horvath, Balint Laszlo Balint

**Affiliations:** ^1^ Department of Biochemistry and Molecular Biology, Genomic Medicine and Bioinformatic Core Facility, University of Debrecen, Debrecen H-4012, Hungary; ^2^ Department of Biochemistry and Molecular Biology, Nuclear Hormone Receptor Research Laboratory, University of Debrecen, Debrecen H-4012, Hungary

**Keywords:** lyophilization, freeze-drying, RNA-seq, biomarker, sustainable biobanking

## Abstract

Biobanks operating at ambient temperatures would dramatically reduce the costs associated with standard cryogenic storage. In the present study, we used lyophilization to stabilize unfractionated human cells in a dried state at room temperature and tested the yield and integrity of the isolated RNA by microfluidic electrophoresis, RT-qPCR and RNA sequencing. RNA yields and integrity measures were not reduced for lyophilized cells (unstored, stored for two weeks or stored for two months) compared to their paired controls. The abundance of the selected mRNAs with various expression levels, as well as enhancer-associated RNAs and cancer biomarker long non-coding RNAs (*MALAT1*, *GAS5* and *TUG1*), were not significantly different between the two groups as assessed by RT-qPCR. RNA sequencing data of three lyophilized samples stored for two weeks at room temperature revealed a high degree of similarity with their paired controls in terms of the RNA biotype distribution, cumulative gene diversity, gene body read coverage and per base mismatch rate. Among the 28 differentially expressed genes transcriptional regulators, as well as certain transcript properties suggestive of a residual active decay mechanism were enriched. Our study suggests that freeze-drying of human cells is a suitable alternative for the long-term stabilization of total RNA in whole human cells for routine diagnostics and high-throughput biomedical research.

## INTRODUCTION

In the past two decades, large collections of biospecimens have been established worldwide in the form of tissue banks and have since become powerful engines of biomedical research. Such biobanks represent invaluable sources of pathological samples for studies with various aims, such as identifying and validating biomarkers or uncovering cellular mechanisms underlying pathological conditions and drug resistance [1–3]. Archiving biological samples in a way that ensures long-term macromolecular integrity and activity provides the opportunity to either rerun diagnostic tests on patient samples weeks or months after collection or enables them to be used in studies operating with large sample sizes for adequate statistical power. Keeping samples stable with consistent quality for long periods is especially important when it is challenging to achieve an appropriate sample size due to the low prevalence of a disease (i.e., rare diseases) [4] or in longitudinal studies.

New trends in biomedical research are relevant for simultaneously profiling hundreds to thousands of genomic sites, RNAs or proteins from blood samples, surgically removed tissue specimens, autopsies or biopsies. The rapidly emerging omic and bioinformatic technologies empower accelerated high-throughput data acquisition and interpretation, translating highly complex genomic, transcriptomic and proteomic data to disease and drug biomarker candidates. Indeed, tremendous effort has been put into screening for biomarkers in a vast number of pathological conditions, utilizing large sample collections and high-throughput technologies, such as whole genome [5–7], exome [8–10] and RNA sequencing (RNA-Seq) [11, 12]. Hence, there is an increasing demand for data-rich samples, especially when no clear future application is defined at the collection phase.

Tissue banks conventionally use cryogenic storage temperatures (generally between –60° C and –180° C) to slow sample deterioration, with substantial operational and environmental costs [1]. These samples are potentially exposed to temperature fluctuations during storage and shipping, which might be detrimental to the integrity of cellular analytes, especially RNA and proteins [13], which are the main targets of current biomarker research due to their dynamic changes in response to various chemical exposures or diseased states, which extend over genetic background-dependent variability. Therefore, introducing a reliable substitute for ultra-low temperature storage would be an important step toward sustainable biobanking. Slowing the enzymatic cleavage of intracellular macromolecules at room temperature can be achieved by chemical fixation or reducing water activity in the sample by dehydration. Formalin-fixed and paraffin-embedded (FFPE) tissue samples can be stored at ambient temperatures for extended periods and are known to preserve cellular and tissue structure well. However, during the various steps of FFPE tissue handling and storage, nucleic acids and proteins become chemically modified and fragmented [14–16], restricting the utility of FFPE samples for molecular biology studies.

Lyophilization (freeze-drying) is a dehydration method in which the samples, usually suspended in a lyoprotectant solution, are quickly frozen and subjected to conditions allowing the frozen water molecules directly enter the gas phase, resulting in a dry end product. Low residual water activity in the final product sufficiently slows sample deterioration, thus extending shelf-life [17]. Moreover, freeze-drying unfractionated cells requires minimal hands-on time and provides the opportunity to preserve multiple heat-labile molecules at the same time, thus maintaining the wide analytical utility of the samples. At present, for the above reasons, freeze-drying is routinely used to preserve food and less complex molecular systems, such as protein preparations (including enzymes, vaccines and antibodies) especially for biotechnological and pharmaceutical purposes [18–20]. In addition, several lyophilization protocols have been established for the long-term maintenance of platelets and living bacterial strains [21, 22].

The most important aspects of a novel, sustainable sample storage strategy include (1) the preservation of data-rich samples such as whole cells and tissues; (2) serving the needs of high-throughput studies by preserving non-fragmented macromolecules; (3) the minimization of sample degradation during storage and shipment; (4) the minimization of associated costs and (5) short hands-on time. Therefore in our study, we tested whether lyophilized human cells would preserve different RNA species over the long-term at room temperature for use in both low- (RT-qPCR) and high-throughput (RNA-Seq) studies. We tested the primary effect of lyophilization as well as the effects of two weeks and two months of room temperature storage in lyophilized form on total RNA yield and integrity and on the performance of low-throughput assays (GAPDH 3′/5′ assay and the transcript-specific RT-qPCR of mRNA, long non-coding RNA (lncRNA) and enhancer-associated RNA (eRNA)). In addition, we sequenced mRNA derived from lyophilized samples that had been stored for two weeks at room temperature to obtain a global view of RNA quality.

## RESULTS

### Quality and quantity of total RNA extracted from lyophilized cells after lyophilization

**Figure 1:**
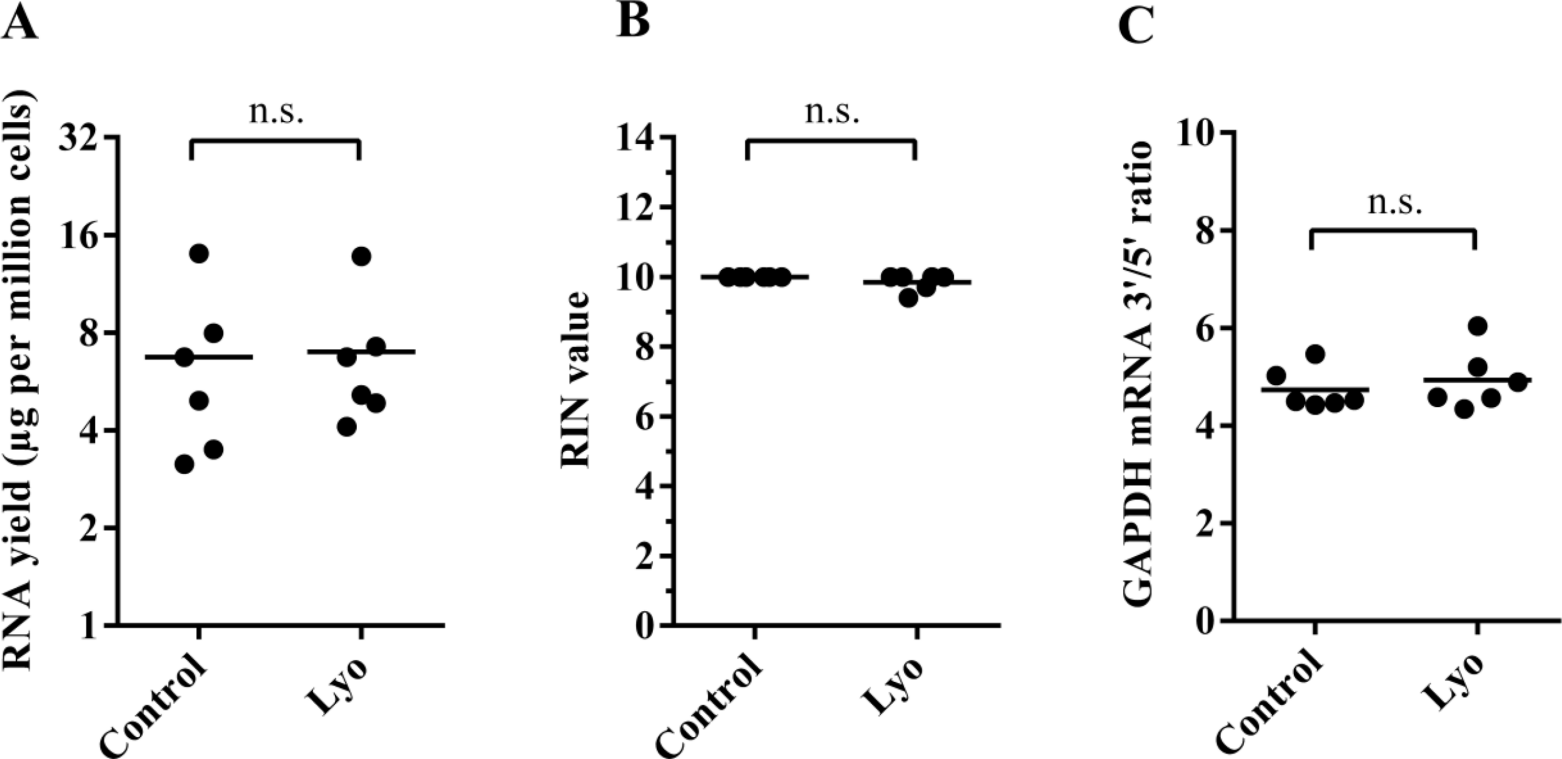
Quantity and quality of RNA isolated from paired control and lyophilized cells (immediately after lyophilization) (**A**) RNA yield per million cells. Horizontal lines represent mean values (*P* = 0.68, paired *t*-test) (**B**) Calculated RIN values of paired samples; horizontal lines indicate mean values (*P* = 0.15, paired *t*-test). (**C**) The GAPDH mRNA 3′/5′ ratio of paired samples (*P* = 0.34, paired *t*-test).

As RNA molecules are inherently labile and sensitive to a number of factors, such as heat, oxidation, pH and especially cellular RNases, we first investigated the effect of our protocol and the freeze-drying cycle itself on the recovery and quality of total RNA extracted from human cells after lyophilization for six hours in 0.1 M trehalose. RNA yields were highly similar between paired non-lyophilized and lyophilized cells when measured immediately after lyophilization (*N* = 6) (Figure 1A). RIN (RNA integrity number) values were calculated from Agilent electropherograms for control samples as 10, while lyophilized samples also showed a remarkably high RIN value average of 9.8 (Figure 1B). Although the RIN value might be an indicator of overall sample quality and is routinely assessed before sensitive gene expression analyses, such as microarrays and RNA-Seq, it has been argued that ribosomal RNA integrity may not reflect that of the mRNA fraction, partly due to structural differences between the two RNA classes [23–25]. Nevertheless, RIN values greater than 7 are generally considered excellent for use in RT-qPCR, microarray and RNA-Seq applications. To investigate mRNA stability directly, we adapted an RT-qPCR-based 3′/5′ integrity assay in which we reversely transcribed total RNA using oligo(dT) priming followed by qPCR amplification of two regions on the *GAPDH* cDNA, one that is located in the 3′ UTR region and another one located ∼1 kb towards the 5′ end, thus deriving a 3′/5′ ratio for each sample; an increased ratio would suggest degradation of the target gene (Supplementary Figure 1). We found no significant difference between the GAPDH mRNA 3′/5′ ratios of controls and lyophilized cells (Figure 1C).

### Lyophilized human cells preserve mRNAs, lncRNAs and eRNAs at different abundances for RT-qPCR

**Figure 2:**
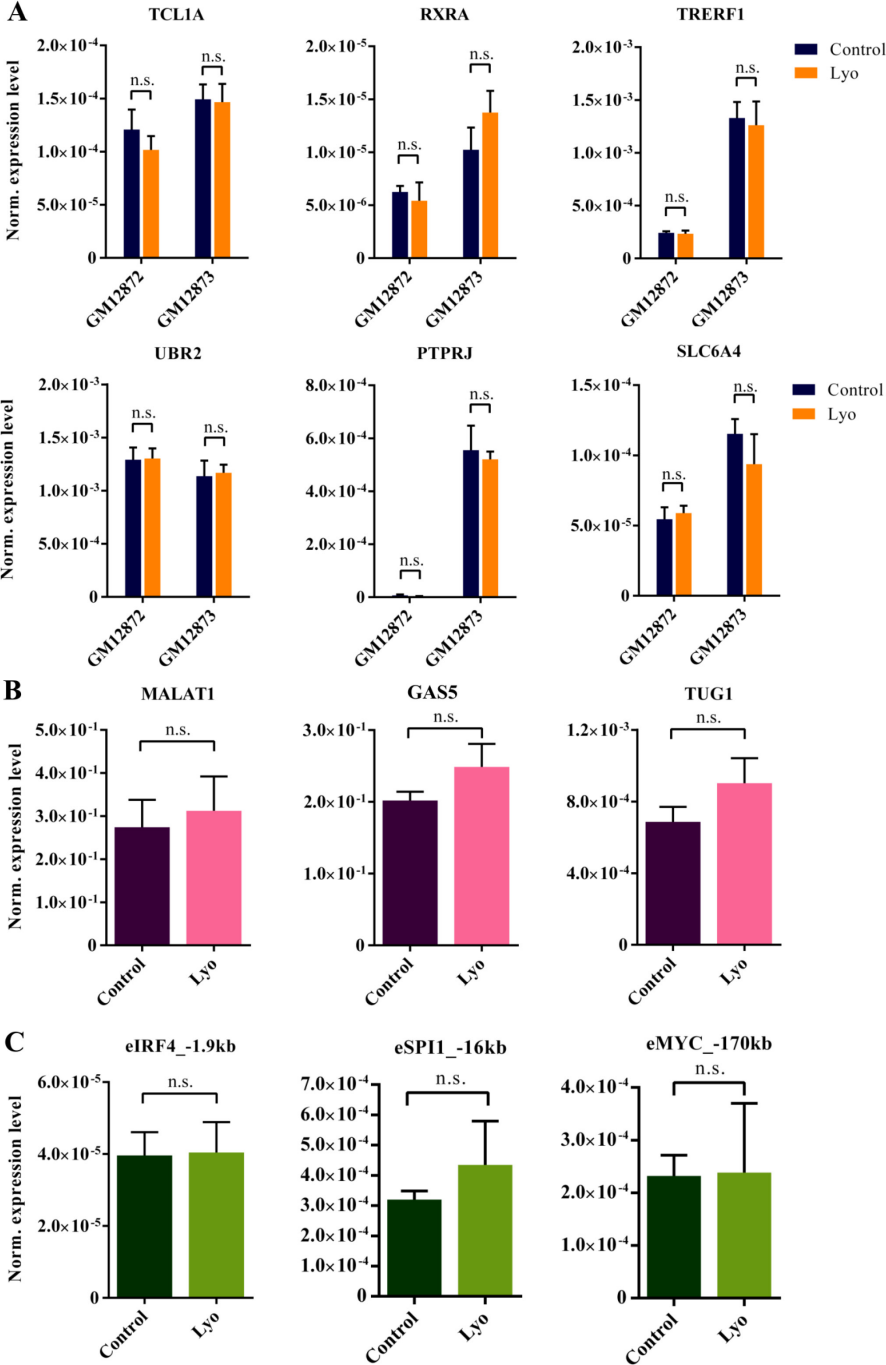
RT-qPCR measurement of mRNAs, lncRNAs and eRNAs with different abundances from total RNA isolated from paired control and lyophilized cells (**A**) Average expression values of genes with different abundances in GM12872 and GM12873 cells normalized to ACTB from paired control and lyophilized samples (error bars represent SEM, *N* = 3). The *P* values were greater than 0.2 in all cases (paired *t*-test) (for values refer to Supplementary Table 4). (**B**) RT-qPCR analysis of selected lncRNAs in paired control and lyophilized GM12873 cells. The ACTB gene was used for normalization. Error bars indicate the SEM, and significance was calculated using a paired *t*-test (*N* = 3). The *P* values for MALAT1, GAS5 and TUG1 were 0.26, 0.25 and 0.43, respectively. (**C**) RT-qPCR analysis of selected eRNAs in paired control and lyophilized GM12873 cells. The ACTB gene was used for normalization. Error bars indicate the SEM, and significance was calculated using a paired *t*-test (*N* = 3). The *P* values for eIRF4_–1.9 kb, eSPI1_–16 kb and eMYC_–170 kb were 0.83, 0.44 and 0.96, respectively.

Because sample degradation might affect mRNAs with different abundances to varying degrees, based on Fragments Per Kilobase Per Million Mapped Reads (FPKM) values obtained from our previous B-Lymphoblastoid cell line (LCL) RNA-Seq data, we measured high (FPKM > 100), moderate (FPKM = 10–100), low (FPKM = 1–10), and extremely low (FPKM < 1) abundance genes in two LCLs: GM12872 and GM12873 (Supplementary Figure 2). Of note, 65% of detectable genes fall into the last two categories. We found that all target genes were amplified, regardless of their abundance, to a similar degree in control and lyophilized cells (Figure 2A). Importantly, this would enable the accurate and bias-free quantification and comparison of differentially expressed transcripts, such as RNA biomarkers, at various expression levels. In recent years, members of the diverse class of long non-coding RNAs have emerged as potentially critical elements of biological regulation, comprising the large majority of the human transcriptome [26, 27]. Being conserved and highly tissue-specific, they have gained considerable attention as potential causative factors in (and as biomarkers of) various diseases, including cancer [28–30]. Although the exact role of the enhancer-associated RNA subclass of lncRNAs is not yet fully understood, these molecules are potent indicators of genomic enhancer activity that might indicate pathological regulatory processes [31, 32]. We selected three lncRNAs of biological relevance (*MALAT1*, *GAS5* and *TUG1*) and three eRNAs associated with super-enhancers of highly expressed LCL genes (*SPI1* – encoding the transcription factor PU.1, *IRF4* and *MYC*). We found high concordance between control and lyophilized samples isolated from GM12873 cells (Figure 2B and 2C).

### Long-term stability of RNA in lyophilized cell powders

**Figure 3:**
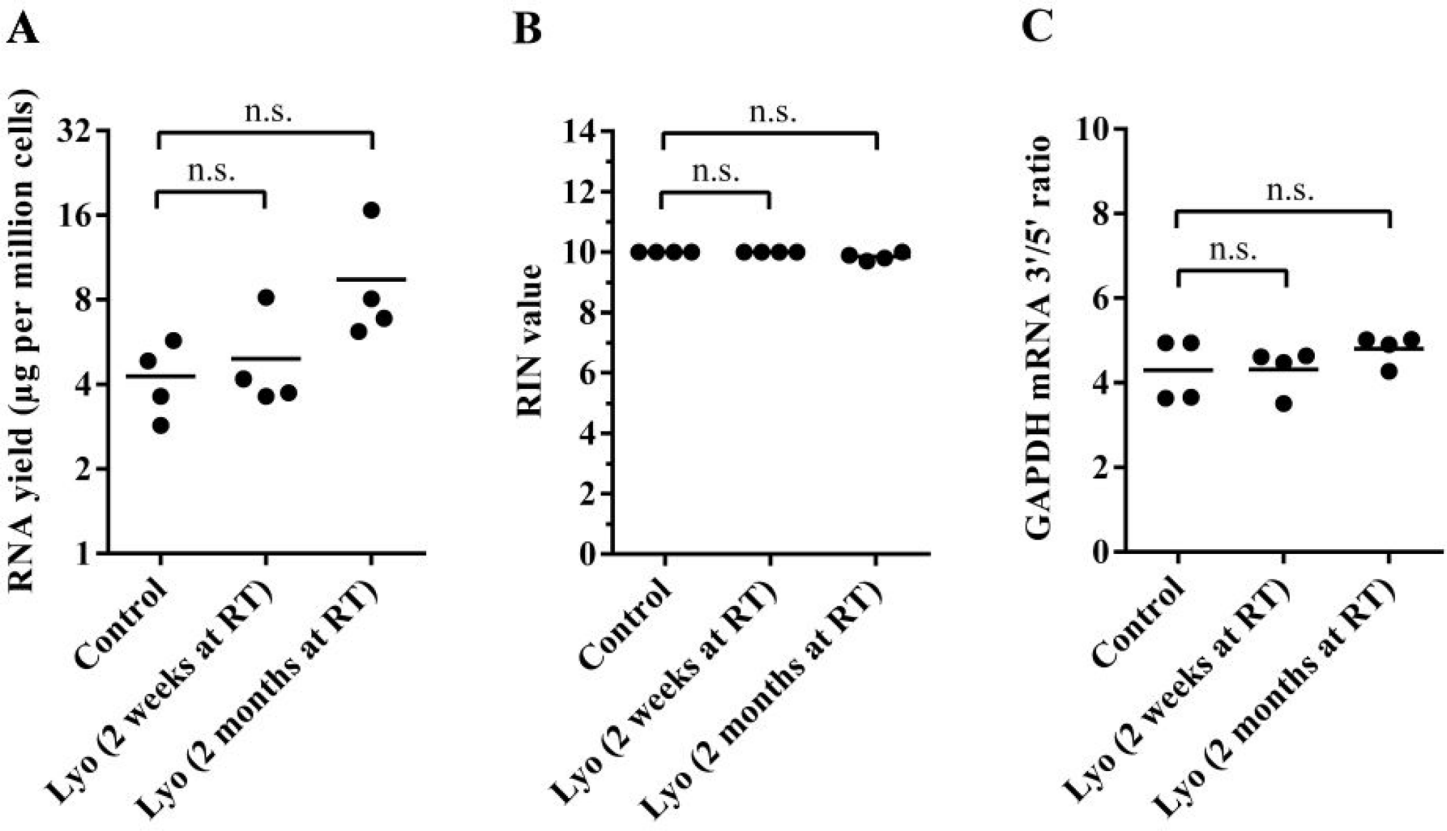
Quality control of total RNA isolated from paired control and lyophilized samples stored for two weeks or two months at room temperature (**A**) RNA yield per million cells. Horizontal lines represent mean values (The *P* value for the two-week samples is 0.97, and that for the two-month samples is 0.16; paired *t*-test) (**B**) Calculated RIN values of paired samples. Horizontal lines indicate mean values (*P* = 1.0 for the two-week samples, and *P* = 0.1 for the two-month samples; paired *t*-test). (**C**) The GAPDH mRNA 3′/5′ ratio of paired samples (*P* = 0.86 for the two-week samples, and *P* = 0.92 for the two-month samples; paired *t*-test).

Various factors may affect the long-term stability of lyophilized cell powders, including heat and light exposure as well as moisture absorption. To avoid sample deterioration due to the factors mentioned above, we stored replicates of lyophilized GM12873 cells in air-tight, dark boxes in the presence of CaCl_2_ dihydrate desiccant at room temperature. We isolated total RNA from lyophilized cells after two weeks and after two months of storage and again compared the yield, RIN values and GAPDH mRNA 3′/5′ ratio between paired control and lyophilized samples. We found no significant difference between paired control and lyophilized cells, indicating that the RNA remains stable in lyophilized cells even after two months of room temperature storage (Figure 3A–3C). We note that during RNA isolation from TRIzolate samples, RNA pellets from two-month-old samples seemed more stable, and sample loss during the washing steps was less evident, which might explain the observed consistent slight, but non-significant increase of RNA yield in those samples.

### RNA-Seq reveals exceptionally high concordance between control and lyophilized samples stored for two weeks at room temperature

Three pairs of total RNA samples isolated from control and lyophilized cell batches that had been stored for two weeks at room temperature were subjected to RNA-Seq library preparation including poly(A) selection and were sequenced on the NextSeq 500 (Illumina) platform (for more details, please refer to the Materials and methods section). The complexity of single-end sequencing libraries can be inferred by calculating quality metrics, such as the fraction of uniquely mapping reads and duplicated reads, which are characteristic of the cell type, library preparation method and the given sequencing run and should be similar within an experiment. Per-sample library information including uniquely mapped reads, read duplication, and the number of detected genes is summarized in Supplementary Table 5. In our dataset, > 90% of sequencing reads mapped uniquely in both control and lyophilized samples, with a slightly higher percentage in lyophilized samples (deviations from the median were between –2.6–0.4%). In high-quality samples, reads with identical start positions occur due to RNA sampling and fragmentation bias, although to a smaller degree, these represent PCR and sequencing artifacts [31]; nevertheless, for RNA of low quality or input quantity, PCR duplicates may dominate the library, leading to decreased complexity. In our libraries, sample deviations from the median duplication rates were between –13.6–6.7%. (Supplementary Table 5). According to Conesa *et al.* [32], samples with less than 30% disagreement for any QC metric are not to be considered outliers; thus, none of our libraries were excluded from subsequent analyses. In summary, the above observations suggest that the RNA-Seq libraries were of high quality and no considerable RNA modifications occurred in the lyophilized samples during either the lyophilization cycle or storage that would affect read mappability and library complexity; these findings contrast with previous reports on FFPE tissue samples, which exhibited higher mismatch rates and decreased mapping quality due to formalin fixation [33, 34].

**Figure 4:**
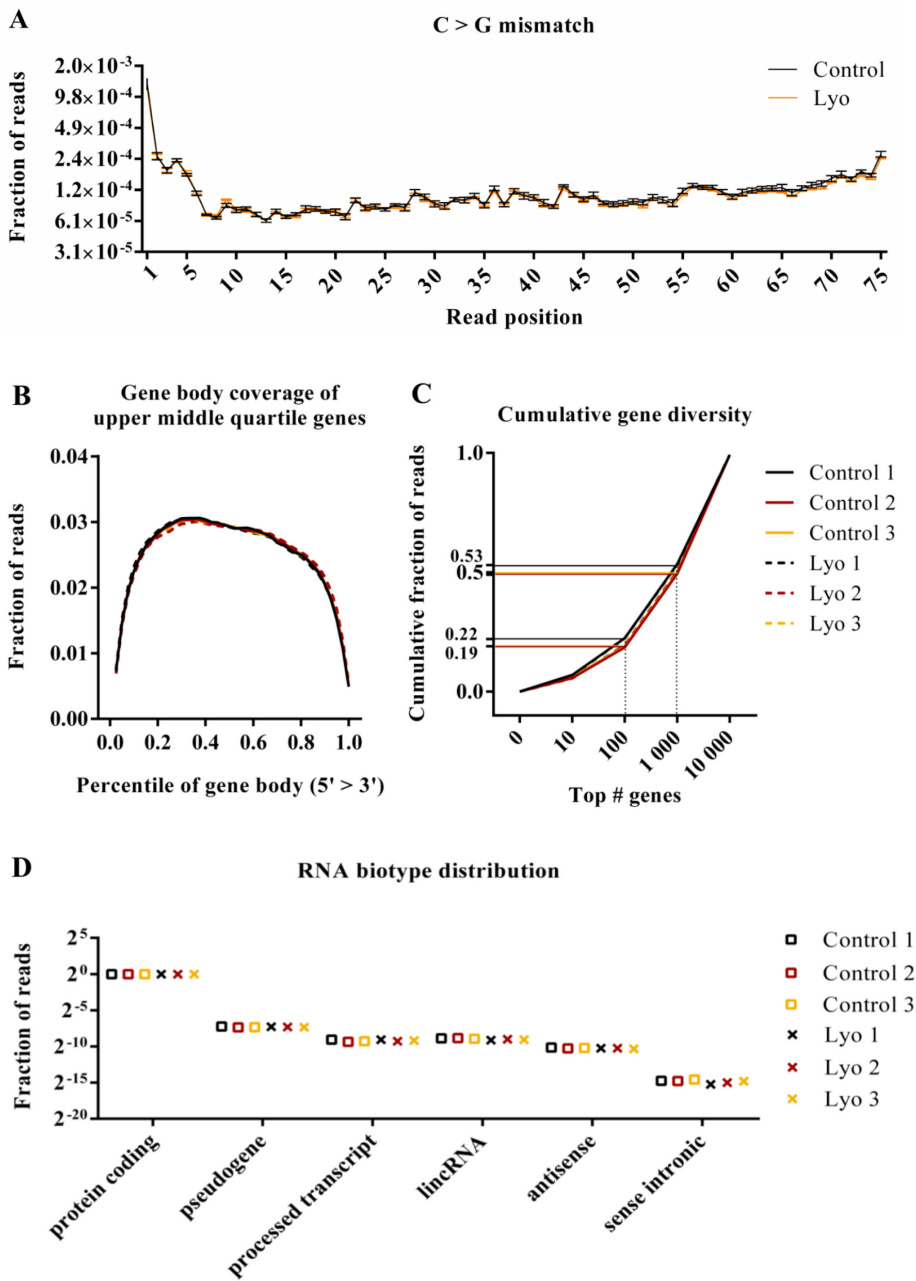
Quality metrics of RNA-Seq data from paired control and lyophilized cells (**A**) Per-base C>G mismatch rate (the mean of three control and three lyophilized samples ± SEM). After correction for multiple testing and at 5% FDR no significant differences were found for any mismatch type at any read position (*P* values > 0.01; *t*-test). (**B**) Gene body coverage profile of genes with read counts in the upper middle quartile range. Each gene’s coding region is divided in percentiles representing 2.5% of the length of the gene, and the fraction of reads mapping to a particular bin is plotted (*P* values < 0.01 for all comparisons; paired *t*-test). (**C**) Cumulative gene diversity. Genes were ranked based on read counts, and the cumulative fractions of reads mapping to the top 10, 100, 1000 and 10000 genes are plotted (*P* values < 0.01 for all comparisons; paired *t*-test). (**D**) The RNA biotype distribution of mapped reads (*P* values > 0.01 for all comparisons; paired *t*-test).

Next, we calculated per-sample information regarding read GC content, per-base mismatch rate, chromosomal distribution, gene body coverage, cumulative gene diversity and RNA biotype distribution (Figure 4 and Supplementary Figure 3). These metrics are used to assess sample- or treatment-specific biases in sequencing libraries. GC plots showed approximately normal distribution, and no sign of contamination or other bias was observed. The plots show a peak between 37–39% GC content (Supplementary Figure 3A). Most reads map to autosomes, with a slight difference between samples; however, the results are consistent within sample pairs, suggesting a cell culture condition-specific difference (Supplementary Figure 3B). We next examined mismatch profiles of the control and lyophilized samples. Reference mismatches partly represent natural variations and may also arise due to the chemical degradation of nucleotides, as for FFPE samples, where G>A and C>T transitions occur. We found no significant difference in mismatch rates between the two groups. One example of this is shown in Figure 4A, and Supplementary Figure 3C shows all types of mismatches represented as ratios between control and lyophilized samples. No difference was found between the control and lyophilized samples in terms of gene body coverage of upper middle quartile genes, suggesting that there was no pronounced 3′ bias due to the 5′ degradation or strand cleavage that is characteristic of low-quality samples (Figure 4B). Calculating cumulative gene diversity can reveal whether an RNA-Seq library is dominated by reads representing a few highly expressed genes, which indicates inferior library complexity. In this case, a characteristic shift towards higher read fractions belonging to the few most abundant genes is expected. Per-sample plots of the libraries show highly similar complexity, with 19–22% of reads occupying the 100 most abundant genes and 50–53% of reads occupying the 1 000 most abundant genes. Although we noticed slightly shifted plots for two samples (Control 1 and Lyo 1), these are paired samples; thus, this phenomenon might reflect the biological condition of the cells at harvest (Figure 4C, 4D) shows the fraction of reads mapping to a certain RNA biotype annotation category per sample. Most reads map to protein-coding genes, and there was no significant difference between the control and lyophilized samples for either of the biotype categories.

### Differentially expressed genes between control and lyophilized samples according to RNA-Seq data

**Figure 5:**
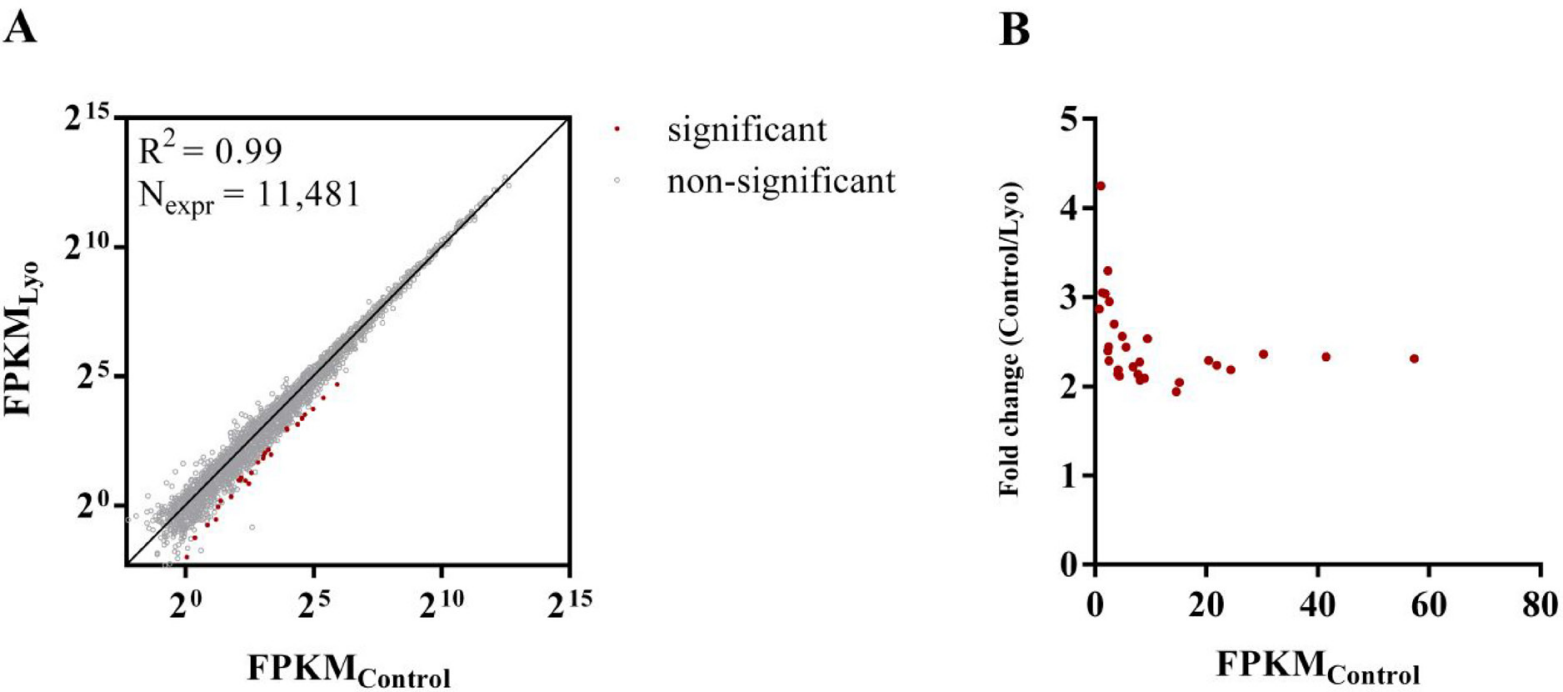
Differentially expressed RNAs in paired control and lyophilized cells (**A**) Log_2_-transformed mean FPKM values obtained from the three lyophilized replicates plotted against those of the controls. Only RNAs with FPKM > 1 in at least one sample of the six were used in the analysis. (**B**) The fold distribution of differentially expressed RNAs as a function of the FPKMs of the controls. Fold values and FPKMs represent the means of three replicates.

We compared the control and lyophilized datasets for differential gene expression and found a high correlation (R^2^ = 0.99) between the mean FPKMs of the control and lyophilized samples. In the lyophilized samples, 28 genes were significantly downsampled at an FDR of 0.05 (Figure 5A). These genes vary by 1.94-4.25-fold (median: 2.31), and the fold change was inversely proportional to the gene expression level in the control cells (Figure 5B). These genes are mostly protein-coding genes (21 protein-coding genes, 6 lowly-expressed lncRNAs with unknown function and 1 pseudogene). We performed Gene Ontology (GO) enrichment analysis on the differentially expressed gene set, and we found that almost half of the protein-coding genes belong to the annotation term ‘DNA-templated transcription’ (GO:0006351; *P* = 2.0^*^10^–5^), with a child term of ‘Transcription by RNA polymerase II’ (GO:0006366; *P* = 2.2^*^10^–5^). These genes encode transcriptional regulators which take part in or modulate transcription initiation, most often by RNA polymerase II, from a DNA template, including the *POLR2A* RNA polymerase receptor subunit, the HMG-box transcriptional repressor *CIC*, the integrator complex subunit *INTS1* and chromatin modifiers *KDM6B* and *KMT2D* (Supplementary Table 6). The overrepresentation of genes encoding transcriptional regulators in the differentially expressed (DE) gene set is consistent with previous studies reporting non-uniform RNA degradation rates in cells cultured under standard conditions or stored at room temperature in aqueous media; transcriptional regulators have been shown to have short half-lives under both physiological and non-physiological conditions [35–39]. This might suggest the presence of some residual activity of regulated cellular RNA decay mechanisms in lyophilized cells, which potentially act on these transcripts based on unique transcript features.

### Evidence of both uniform and non-uniform read distribution over the gene bodies of differentially expressed transcripts between control and lyophilized samples

**Figure 6:**
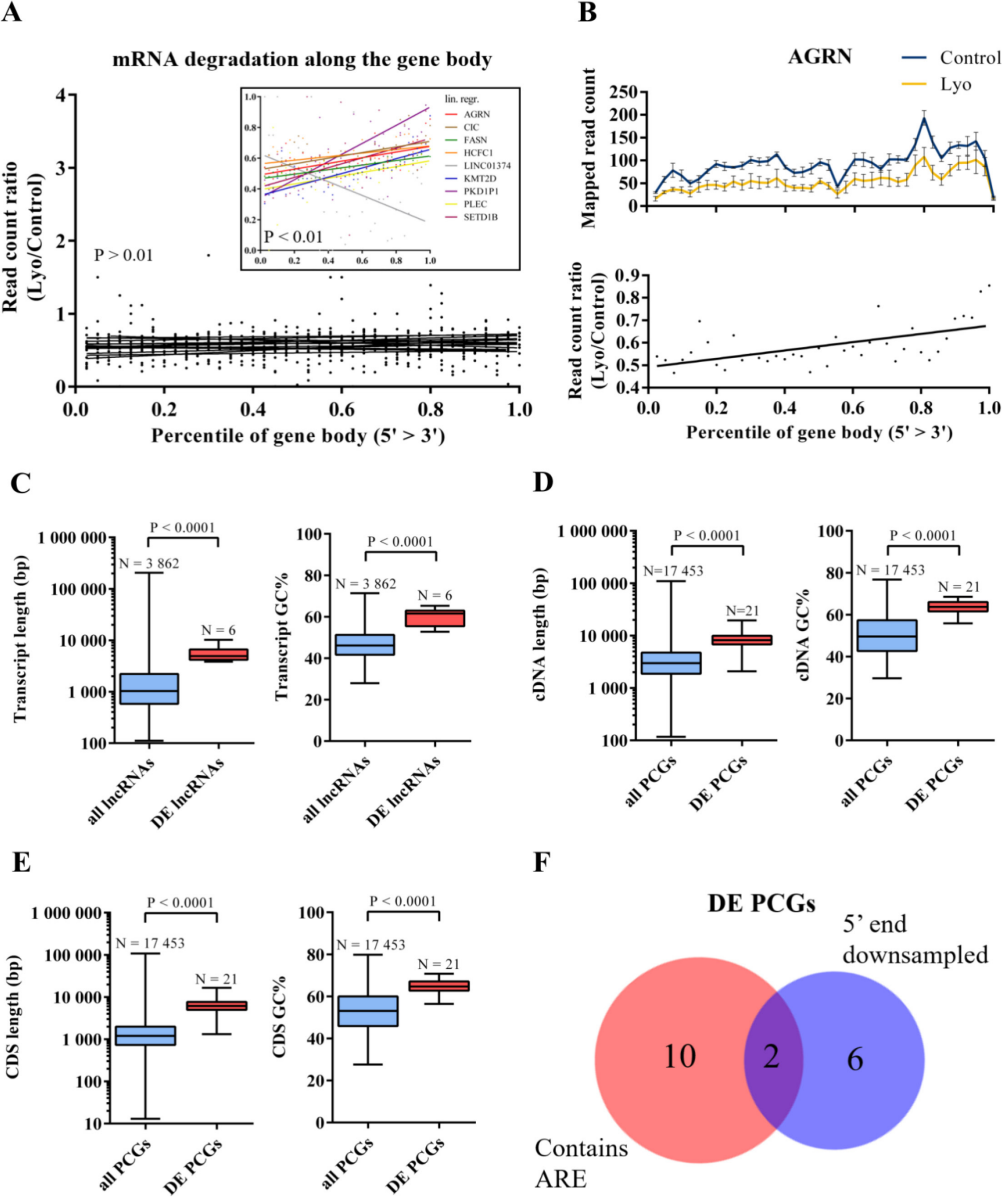
Transcript properties of differentially expressed RNAs (**A**) Read count ratios across 40 bins of the meta-transcripts assembled from all exonic regions for the 9 differentially expressed RNAs with 5′ or 3′ degradation bias (linear regression analysis; *P* < 0.01). (**B**) Mapped read counts in control vs lyophilized samples (mean ± SEM) and read count ratios across the *AGRN* metatranscript. A linear regression curve was fitted to the read count ratio data. (**C**) Box-and-whisker plots are showing transcript lengths and GC counts of all human versus differentially expressed lncRNAs. (**D**) Box-and-whisker plots showing cDNA lengths and GC counts of all human versus differentially expressed protein-coding RNAs. (**E**) Box-and-whisker plots showing CDS lengths and GC counts of all human versus differentially expressed protein-coding RNAs. All box-and-whisker plots display medians (horizontal line) and interquartile ranges (box) and minimum to maximum values (whiskers). Gene numbers and *P* values are indicated. (**F**) Venn diagram of differentially expressed RNAs containing ARE(s) and/or showing significantly biased degradation of the 5′ end (*P* < 0.01). DE = differentially expressed; PCG = protein-coding genes.

Next, we sought to investigate whether the 5′ and 3′ ends of these DE RNAs are affected by degradation to differing degrees. RNA degradation by fragmentation lead to the underrepresentation of 5′ ends in sequencing libraries utilizing poly(A)-capture, as cleaved 5′ ends (without poly(A) tract) are washed off from the capture beads, therefore only the 3′ end of fragmented transcripts will be processed through the subsequent steps of the library preparation protocol. First, we counted the reads mapping to each of the 40 bins defined across meta-transcripts (containing all exonic regions belonging to the given DE genes) for each sample. Read counts belonging to the same sample group were then averaged in each bin, and a read count ratio was derived bin-to-bin by dividing the average read counts in lyophilized samples by the average read counts in controls. We found that for most DE genes the read count ratio did not correlate with the downstream distance from the 5′ end (the slope of the linear regression curve is non-significantly different from zero, *P* > 0.01; *N* = 16); 8 DEGs show significant positive and 1 DEG (the lowly abundant *LINC01374;* in controls, mean FPKM is as low as 1.04, and more than half of the first 20 bins contain zero or 1 read) shows significant negative correlation between the two variables (the slope of the linear regression curve is significantly above or below zero, respectively; *P* < 0.01). (Figure 6A). From the above analysis, we can conclude that for the majority of DE transcripts the number of reads is uniformly reduced across the gene body; however, in one-third of the genes, the 5′ end is underrepresented in the sequencing library, which suggests that RNA fragmentation might contribute to RNA degradation observed in lyophilized samples. Figure 6B shows the read count ratio distribution over the meta-transcript of the *AGRN* gene as an individual example.

### Certain transcript properties correlate with differential gene expression between control and lyophilized samples

Next, we asked which transcript properties might be associated with RNA decay in lyophilized samples (for analysis details see Materials and methods; Supplementary Table 7). We found that the median transcript length and %GC content were significantly higher in DE lncRNAs and protein-coding RNAs (5′UTR+CDS+3′UTR) compared to all human lncRNAs and protein-coding RNAs, respectively (*P* < 0.0001 in all cases, Mann-Whitney test; Figure 6C–6D). We also examined the length and %GC content of the coding DNA sequence (CDS), the 5′UTR and 3′UTR of protein-coding RNAs, and we found that the length of the CDS, as well as the %GC content of the CDS, the 5′UTR and 3′UTR were significantly higher in the DE set (*P* < 0.001 in all cases, Mann-Whitney test; Figure 6E and Supplementary Figure 4A, 4B). It is now well established that AU-rich sequence elements (AREs) in the 3′UTR are associated with shorter RNA half-life through an active decay mechanism mediated by ARE-binding proteins predominantly through facilitating deadenylation of the poly(A) tail under physiological conditions in mammals [40]. Based on the ARED-Plus database [41], 12 of the DE protein-coding genes (57%) have transcripts with single or multiple ARE elements in the 3′UTR and/or in intronic regions (Supplementary Table 7). Notably, there is only a modest overlap between genes with AREs and genes with non-uniform downsampling of the two transcript ends, suggesting that DE protein-coding genes decay either by fragmentation or ARE-mediated decay (Figure 6F).

## DISCUSSION

A rapidly growing number of studies suggest that both intra- and extracellular RNA molecules are able to serve as diagnostic and prognostic markers for various diseases, including cancer [42]. Unlike DNA biomarkers, RNA expression is highly dynamic and reflects the functional state of a biological system, mirroring both genetic and epigenetic gene regulatory mechanisms. Furthermore, the high specificity and sensitivity of RNA detection methods make them a more attractive alternative than protein biomarkers. The rapidly decreasing costs of RNA sequencing and the expanding number of bioinformatics tools have enabled researchers to take a global view on differentially regulated pathways between tissues from control versus diseased patients, which has led to the construction of a number of RNA-based biomarker panels, including the PAM50 breast cancer subtype predictor panel [43]. Maintaining large and cost-effective biorepositories, as well as facilitated sample sharing, will serve this new era of biomarker research generating high-throughput gene expression profiles.

The current standard practice for preserving native biological samples for molecular analyses is flash-freezing followed by cryogenic storage in liquid nitrogen or in deep freezers. There are two major drawbacks associated with biobanks relying on ultra-deep temperatures: emerging concerns over financial sustainability due to substantial running costs, shrinking funding resources and the risk of transient warming cycles due to, for instance, power outages or suboptimal transport conditions, which may result in sample deterioration seriously affecting RNA integrity [1, 13]. Cross-border transfers from large international biobanks are especially detrimental due to logistical barriers and long delays. Therefore, there is an increasing need for biospecimen storage and transportation at ambient temperatures.

Short-term RNA stabilization in tissues at non-cryogenic temperatures using RNAlater and other cell-penetrable fixatives (e.g. PAXgene and Allprotect) which precipitate cellular RNases has become widely used in the past few years, especially when the availability of dry ice and liquid nitrogen for flash-freezing is restricted [44, 45]. However, it is generally recommended to freeze the samples after up to one week or one month of storage at room temperature or between 4–8° C, respectively. Moreover, although RNAlater has been successfully used for short-term tissue storage before microarray analyses [46, 47], a recent report on the systematic effect of RNAlater on the transcriptome and proteome of plant cells warrants caution when using RNAlater stabilization prior to high-throughput studies [48].

Well-annotated, archival FFPE tissues stored for extended periods of time at room temperature have been increasingly recognized as a potentially rich source of molecular information for medical research. However, as the fixation of cellular structures and tissue morphology for microscopic evaluation often takes priority over the preservation of intact biomolecules, extensive nucleic acid and protein deterioration occur during fixation, embedding, storage and tissue isolation, seriously limiting the usefulness of FFPE samples in studies utilizing high-throughput omic technologies [14, 15, 49, 50]. The addition of hydroxymethyl groups during formalin fixation to all four nucleobases, especially adenine, followed by methylene bridge formation between two neighbouring amino groups has been shown to impair both random hexamer- and oligo(dT)-primed reverse transcription, as well as subsequent PCR amplification [51–53]. Additionally, RNA in FFPE tissues is prone to hydrolysis due to various factors during storage, deparaffinization and crosslink reversal, generally resulting in short RNA fragments, leading to the underrepresentation of 5′ ends of the transcripts in sequencing libraries [34]. Furthermore, during paraffin embedding, G>A and C>T substitutions emerge in nucleic acids due to nucleobase deamination, possibly leading to read mapping bias and hindering reliable SNV calling from DNA and RNA sequencing data [33, 34, 48, 49, 54, 55].

Lyophilization has emerged as an alternative preservation method for biologicals and is already widely used by the pharmaceutical and food industries to increase the shelf-life of therapeutics and food. A vast number of publications from the past decades have shown that living bacteria and yeast cells can be recovered after freeze-drying. Also, there is considerable interest in using lyophilized whole animal cells (including human) in assisted reproduction and regenerative medicine. However, lyophilizing these delicate cells in a way that their viability is retained upon rehydration generally requires more resource-intensive experimental design, including loading combinations of protectants into the cells and using tightly controlled freezing, drying, storage and reconstitution conditions. Platelets have long been lyophilized and were shown to have high functional recovery rates, especially when loaded with trehalose [56]. However, only a few studies reported successful lyophilization and subsequent recovery of membrane-intact, functional nucleated cells so far [57, 58]. Strikingly, freeze-dried, non-viable nucleated cells have also proven sufficient for certain downstream applications. An early study showed that injecting heads of lyophilized dead sperm cells into oocytes leads to normal embryonic development [59]; furthermore, nuclei of freeze-dried somatic cells were used with success in somatic cell nuclear transfer [60], suggesting that cell viability is not necessary for some downstream applications.

Several studies have been conducted to assess the analytical utility of various freeze-dried tissues, the majority of which used electrophoretic methods and traditional RT-(q)PCR of one or a few selected genes as measures of RNA integrity. Lyophilization protocols varied substantially in excipient use and drying length. Early studies by Takahashi *et al.* and Matsuo *et al.* [61, 62] showed that rat tissues lyophilized for 2 hours in hexene and stored for four years were similar to their fresh-frozen counterparts using various small-scale techniques. However, not surprisingly, RNA degradation was more prominent and rapid than that of DNA and proteins. Mareninov *et al.* [63] lyophilized brain tumour tissues without excipients for 72 hours and draw similar conclusions in terms of RNA stability after one year of room temperature storage. Leboeuf *et al.* [64] lyophilized both tissue segments and cell lines without excipients for 29 hours. They have found that the most important factors of RNA stability during storage were light protection and the presence of desiccants, however, surprisingly, room temperature storage was found to be slightly superior to storage at 4° C in terms of RNA integrity (RIN values and qPCR signal of selected mRNAs). The long-term stability of RNA in lyophilized tissues is probably dependent on the size of the tissue section, as the efficient removal of water molecules from the middle part of a thick tissue slice would be hindered by the upper cell layers, possibly increasing drying time. Also, it has been shown that lipid peroxidation in dried samples mediates nucleic acid degradation [65]. Thus the introduction of antioxidants into lyophilization formulations might aid in preventing oxidative degradation of dried tissues in the long term. Dry preservation of various biofluids, for example, tear and cerebrospinal fluid for RNA analysis would require unique factors to be considered, such as the volume required for optimal detection sensitivity and their composition, which essentially differ from that of the intracellular space. However, no articles have been published yet on the stability of RNA in lyophilized biofluids. Although freeze-drying of whole blood has been used for preserving genomic DNA for HLA typing [66], analyzing circulating or cellular RNA would require the separation of blood constituents prior to lyophilization, as possible cell membrane leakage induced during cell recovery might lead to cross-contamination of the sample fractions.

In case of cell viability is not a requirement, a cost-effective, reasonably quick and less stringent lyophilization protocol would be desirable. Our primary goal was to apply a technique for freeze-drying RNA in the cellular context that does not compromise between high RNA quality and low cost. Also, preserving unfractionated cells may provide a wide analytical utility to the samples by preserving the most important cellular analytes (DNA, RNA and proteins) in a dried state over the long term at room temperature. Thus, we flash-froze whole LCLs resuspended in a simple 0.1 M trehalose/PBS solution without allowing time for trehalose to load into the cells avoiding the possible perturbation of the steady-state transcriptome [67, 68]. Then, using a manifold freeze dryer, where the heat needed for drying was transferred to the product primarily through convection and radiation from the surrounding laboratory environment (conditioned to 22° C), we could substantially shorten drying time, down to six hours, compared to shelf freeze dryers. Drying time is an important factor to consider as per-sample lyophilization costs mostly depend on the energy consumption of the freeze dryer. Although reports suggest that pharmaceutical elegance of the dried product (the so-called “cake” structure) might not be a good predictor of sample quality [69, 70], in the pharmaceutical industry cycle optimization efforts are mostly aimed at obtaining products with stable cake structure; therefore lyophilization cycles often take 24 to 120 hours depending on the type and operation protocol of the freeze dryer, sample formulation, volume and surface area. At the end of our freeze-drying procedure trehalose formed a dry collapsed matrix around cells, from which membrane-intact cells couldn’t be recovered probably due to cell membrane damage during freezing, drying, or rehydration. Although the dried product did not preserve the classical cake structure, the powder was easily dissolvable in TRIzolate even after long-term storage, and total RNA could be isolated at high quality and quantity. These observations suggest that neither time-consuming trehalose loading nor elaborate and long drying cycles to preserve intact cell membranes and classical cake structure are a requirement for preserving highly intact RNA in dried cells. We aimed at storing lyophilized cells at room temperature ensuring conditions previously described as advantageous for dry storage; i.e., in the presence of desiccants to prevent moisture absorption and protected from light. As TRIzolate practically enables the simultaneous isolation of DNA, RNA and proteins, future studies are warranted to assess DNA and protein integrity using a similar lyophilization technique.

We applied fluorometry and microfluidic electrophoresis for the initial assessment of total RNA quantity and quality isolated right after lyophilization and after long-term storage at room temperature. However, residual moisture content and room temperature storage might pose a risk of physical and chemical deterioration for RNA molecules that might not have been captured by using electrophoretic methods. We found that normalized expression levels of selected mRNAs, including mRNAs of extremely low expression, and long non-coding RNAs that have been identified as potential biomarkers of various diseases did not differ between control and lyophilized samples when assessed by RT-qPCR. Enhancer-associated RNAs have been under intensive research in the recent years for their potential role in gene expression regulation, and to date, no other publications assess the stability of this RNA type in lyophilized cells. In our experiments, similarly to mRNAs and lncRNAs, the three assessed eRNAs showed no significant abundance change upon lyophilization. By using a 3′/5′ assay involving oligo(dT)-primed RT reactions and subsequent qPCR-based quantitation of two regions ∼ 1 kb apart from each other along the *GAPDH* mRNA, we could show that there was no pronounced 3′ bias for this gene in lyophilized samples, suggesting that no considerable strand cleavage occurred between the *GAPDH* mRNA regions assayed.

It cannot be generalized that RNA samples performing well in PCR-based applications will enable robust transcript quantifications using RNA-Seq. Although several studies have concluded that (q)PCR-based gene expression estimates are relatively insensitive to overall RNA quality, especially when random hexamer primers are used for reverse transcription and when amplified regions are short [38, 71, 72], significant gene expression changes have been reported using high-throughput methods [73, 74]. Therefore, and as large-scale biomarker screens mostly utilize RNA-Seq to obtain a global view on disease- or treatment-specific gene expression changes, we sequenced RNA samples from lyophilized, two-week samples and their matched controls to get a global picture of transcriptome changes specific to stored, lyophilized samples that might not have been captured using low-throughput methods. Although we found largely uniform sequencing library properties (uniquely mapped reads, read duplication rates, number of detected genes, GC%, library complexity, gene body coverage, and read distribution over different chromosomes and across various RNA biotypes), as well as no signs of modifications affecting base detection across all samples, we found 28 genes that were significantly downsampled in lyophilized samples, though with a low median fold-change. Although our differentially expressed set is small, we found evidence that RNA fragmentation and residual activity of regulated decay mechanisms might play a role in their degradation.

The lyophilization protocol might be improved by increasing trehalose concentration, as the higher the trehalose concentration is, the higher the glass transition temperature becomes, allowing for lyophilization and storage at higher temperatures; notably, long exposure times to trehalose during fluid-phase trehalose loading into cells might lead to specific transcriptome changes, which should be avoided if lyophilized cells are to be used for RNA studies [67, 68]. Also, setting up a freeze-drying cycle targeting a preset residual moisture content of the final product to a level that minimizes biological activity, while not hampering RNA isolation, would help further increase reproducibility after long-term storage. Furthermore, the lyophilization solution may be supplemented with additives such as antioxidants or other chemicals to improve cell membrane stability, enabling whole-cell experiments, such as flow cytometry or chromatin immunoprecipitation.

Taken together, the findings of our study provide information about the feasibility of lyophilization for the preservation of total RNA in human cells for both low- and high-throughput studies. Introducing lyophilization to the practice of clinical sample preservation would aid in setting up economic and safe large tissue repositories of data-rich samples to satisfy the needs of the post-genomic era.

## MATERIALS AND METHODS

### Cell culture

EBV-transformed B-lymphoblastoid cell lines of the HapMap pedigree 1459 (GM12872 and GM12873) were obtained from Coriell Cell Repositories and were cultured according to the supplier’s guidelines. Briefly, cells were seeded at a concentration of 2^*^10^5^ cells/ml in RPMI-1640 with sodium bicarbonate (Sigma-Aldrich, cat. R0883) supplemented with 15 v/v% heat-inactivated FBS (Gibco, Thermo Fisher Scientific, cat. 10270-106), 2 mM L-glutamine (Sigma-Aldrich, cat. G7513) and 1 v/v% penicillin-streptomycin (Sigma-Aldrich, cat. P4333). Cells were harvested for experiments at subconfluence (up to 8^*^10^5^ cells/ml).

### Lyophilization

Three million cells were washed with PBS and resuspended in 0.5 ml of lyophilization solution which was 0.1 M D-(+)-Trehalose dihydrate (Sigma-Aldrich, cat. T9531) in PBS. Cell suspensions were placed in safe-lock polypropylene microcentrifuge tubes and were snap-frozen by immersing sideways in liquid nitrogen. Immediately before loading into the freeze dryer, the tubes were opened, and a film in which 7 holes were pierced (1 mm diameter each) was placed on top of the tube’s opening. The samples were loaded into a CoolSafe 110 freeze dryer (ScanVac, LaboGene, Denmark) belonging to the Proteomics Core Facility at the University of Debrecen, Hungary, which has a condenser temperature as low as –110° C and a vacuum pump capable of reaching an absolute pressure of 0.004 mBar. Samples were lyophilized for 6 hours (with the environmental temperature conditioned to 22° C) and were either processed immediately or stored for 2 weeks or 2 months at room temperature (23–25° C) in the presence of CaCl_2_ dihydrate (desiccant) in a non-transparent, tightly sealed box to prevent moisture absorption and light exposure.

### Total RNA isolation and basic quality control

Fresh cell pellets or lyophilized powders containing 3 million cells were carefully resuspended and vortexed for 5 min in 1 ml TRIzolate reagent (UD-Genomed Medical Genomic Technologies Ltd., cat. URN0103). Phase separation was carried out using chloroform (1:5) (Sigma-Aldrich, cat. C2432) and high-speed centrifugation. RNA was precipitated from the aqueous phase for 10 min at room temperature using isopropanol (1:1) (Sigma-Aldrich, cat. I9516). Pellets were washed twice with chilled 75% ethanol (diluted with nuclease-free water from absolute ethanol, VWR International, cat. 20821.296), vacuum-concentrated and redissolved in nuclease-free water (AccuGENE, Lonza, cat. 51200) at 65° C for 10 min. Sample purity was determined using a NanoDrop 1000 instrument (Thermo Fisher Scientific, Waltham, MA, USA), and accurate concentrations were determined using a Qubit RNA HS Assay Kit (Thermo Fisher Scientific, cat. Q32855). Each RNA sample was loaded on Agilent RNA 6000 Nano microchips (Agilent, Santa Clara, CA, USA) according to the manufacturer’s recommendations for the analysis of total RNA fragment distribution and calculation of RIN values.

### RT-qPCR

For the 3′/5′ GAPDH mRNA integrity assay, we used the forward and reverse primers from Sigma-Aldrich’s 3′/5′ assay system, which amplify a portion of the 3′ UTR (‘3′GAPDH’) and a region approximately 1 kb upstream (‘5′GAPDH’) in the human GAPDH mRNA. For mRNA RT-qPCR assays (UBR2, TRERF1, PTPRJ, SLC6A4, RXRA and TCL1A), qPCR primers were designed using the UPL Assay Design Center (Roche Applied Science, Germany) making use of cell-specific alternative transcript information (generated from our untreated GM12872 and GM12873 RNA-Seq data with Cufflinks). Primer 3 Plus (http://www.bioinformatics.nl/cgi-bin/primer3plus/primer3plus.cgi) was used to design eRNA and lncRNA primers. For eRNA primer design, we utilized our in-house LCL H3K27ac ChIP-seq and mRNA-seq data (unpublished), as well as public polII ChIA-PET and GRO-cap data from GM12878 LCL (GEO accession numbers GSM1872887 and GSM1480323). For primer sequences used in this study, see Supplementary Tables 1–3.

For the 3′/5′ GAPDH mRNA assay and lncRNA and eRNA measurements, 1-1.5 μg total RNA was treated with RQ1 DNase (1 unit/µg total RNA) according to the manufacturer’s specifications prior to reverse transcription (Promega, cat. M6101). Total RNA (2.5 ng for the 3′/5′ assay, 250 ng for lncRNAs and eRNAs and 500 ng for mRNAs) was reversely transcribed using the SuperScript II system (Thermo Fisher Scientific, cat. 18064014), including 1x FS buffer, 10 mM DTT, 0.5 mM dNTP mix, 0.8 U of SSII enzyme supplemented with 0.4 µg oligo-p(dT)15 primers (3′/5′ assay), 100 nM gene-specific RT primers (lncRNA and eRNA) or 0.012 µg random hexamer primers (mRNA). In the case of lncRNA and eRNA measurements, the protocol was performed in two separate steps: DNase-treated total RNA samples were first incubated at 65° C for 5 min with 1 mM dNTPs and 200 nM gene-specific primers in a 5-µl final volume and chilled on ice; then the preincubated samples were resuspended with a master mix (5 µl; 5x FS buffer, DTT, SSII enzyme and nuclease-free water) to reach final concentrations of reagents described above. The thermal profiles for each RT protocol were as follows: 42° C for 2 hours, 70° C for 15 min (3′/5′ assay), 42° C for 50 min, 70° C for 15 min (lncRNAs and eRNAs), and 25° C for 10 min, 42° C for 50 min and 70° C for 15 min (mRNAs). Control reactions lacking reverse transcriptase were prepared for each sample.

RT reactions including controls were diluted five-fold with nuclease-free water and subsequently subjected to singleplex qPCR (in a 10-μl final volume) using the LightCycler 480 SYBR Green I Master (Roche Applied Science, cat. 04887352001) with 0.375 µM of each of the forward and reverse primers. The cycling parameters were 95° C for 10 min, followed by 50 cycles of 95° C for 5 s, 55° C for 15 s and 72° C for 10 s (‘3′ GAPDH’ and ‘5′ GAPDH’ primer pairs), 95° C for 10 min, followed by 50 cycles of 95° C for 10 s and 60° C for 30 s (lncRNAs, eRNAs and mRNAs). The qPCR measurements were carried out in triplicates for each data point. The GAPDH mRNA integrity ratio was calculated using qPCR efficiency corrected Cp values obtained from the ‘5′GAPDH’ and ‘3′GAPDH’ measurements for a given sample (2^-ΔCp^). Expression levels of lncRNAs, eRNAs and mRNAs were quantified using the ΔCp method and were normalized to *ACTB* expression. On analyzing the melting curve profiles, we observed single amplicons at the expected Tm values.

### RNA-Seq library preparation and sequencing

Sequencing libraries were prepared following Illumina’s TruSeq RNA Sample Preparation v2 Guide with poly(A) selection using 1 μg total RNA as the starting material. Indexed libraries were pooled and subjected to single-end sequencing to an average depth of ∼23 million reads on a NextSeq 500 sequencer with 75-bp read length (Illumina, San Diego, CA, USA). Library preparation was performed at the Genomic Medicine and Bioinformatic Core Facility at the University of Debrecen, Hungary, while cluster generation, sequencing and base calling were performed at the Prof. Balázs Győrffy laboratory at the 2^nd^ Department of Pediatrics, Semmelweis University, Budapest, Hungary. Demultiplexing was performed using the bcl2fastq Conversion Software (Illumina).

### RNA-Seq data analysis

Sequencing reads were aligned to the hg19 genomic build (GRCh37) with TopHat v2.0.7. keeping reads mapping only to one genomic position (—max-multihits option set to 1). Mapped reads were sorted with SAMtools and Picard’s MarkDuplicates was used for flagging and counting duplicated reads. Transcript abundances were calculated using Cufflinks and are expressed as FPKM values. Genes with FPKM values below 1 across all samples were considered unexpressed and were discarded, as were poly(A)-free small RNAs (snRNAs, snoRNAs, scaRNAs, vtRNAs snaR genes and miRNAs) due to ambiguous capture during poly(A) selection. We used Cuffdiff at an FDR of 0.05 without fold restriction to find genes that were differentially expressed between the control and lyophilized sample groups (mean FPKM > 1 in at least one group). The QoRTs package [75] was used to generate metadata regarding GC content, per-base mismatch profile, chromosome distribution, absolute read count per gene and gene body coverage (across bins of meta-transcripts representing non-overlapping genes). Cumulative gene diversity was calculated for each sample by sorting the genes based on read count and plotting the fraction of reads mapping to the top 10, 100, 1 000 and 10 000 genes. RNA biotype assignment of the genes with available HGNC ID (retrieved from https://www.genenames.org/) was performed using ENSEMBL v92 annotation. The DAVID Bioinformatics Resources 6.8 tool was used for the functional annotation of differentially expressed genes (https://david.ncifcrf.gov/). For transcript feature calculations, the longest transcript variant of human lncRNAs and longest transcript variants of protein-coding genes with available CDS were downloaded from the HGNC database (ftp://ftp.ebi.ac.uk/pub/databases/genenames/new/tsv/locus_types/). Transcript, CDS and UTR sequences were retrieved using ENSEMBL v91 genes from BioMart. Sequence lengths and %GC contents were calculated from transcript sequences using a custom bash script (awk). To accept or reject the null hypothesis that the median values are not significantly different between all vs differentially expressed genes, we used the two-tailed non-parametric Wilcoxon rank-sum test (Mann–Whitney *U* test), which accounts for the different sample sizes. ARE data for the differentially expressed set were retrieved from the ARED-Plus database [41]. BioVenn was used to draw a Venn diagram [76].

### Statistical analysis and visualization

Statistical tests of the data presented and all data visualizations throughout the paper were performed using GraphPad Prism version 6.01 for Windows, GraphPad Software, La Jolla California, USA; www.graphpad.com.

### Data access

The RNA-Seq data have been deposited in the GEO database under accession GSE106344.

## SUPPLEMENTARY MATERIALS FIGURES AND TABLES



## Abbreviations

ARE: AU-rich sequence element
CDS: coding DNA sequence
DE: differentially expressed
dNTP: deoxynucleotide triphosphate
DTT: ditiotreitol
eRNA: enhancer-associated RNA
FBS: foetal bovine serum
FDR: false discovery rate
FFPE: formalin-fixed and paraffin-embedded
FPKM: fragments per kilobase per million mapped reads
GSP: gene-specific priming
LCL: B-lymphoblastoid cell line
lncRNA: long non-coding RNA
lincRNA: long intergenic non-coding RNA
Lyo: lyophilized
miRNA: microRNA
PBS: phosphate-buffered saline
QC: quality control
RIN: RNA Integrity Number
RNase: ribonuclease
RNA-Seq: RNA sequencing
RT(1): room temperature
RT(2): reverse transcription
RT-qPCR: quantitative reverse-transcription polymerase chain reaction
scaRNA: small Cajal body-specific RNA
snaR: small NF90-associated RNAs
snoRNA: small nucleolar RNA
snRNA: small nuclear RNA
SSII: SuperScript II
SNV: single nucleotide variant
UPL: Universal ProbeLibrary
UTR: untranslated region
vtRNA: vault RNA
