# Correction: Lyophilized human cells stored at room temperature preserve multiple RNA species at excellent quality for RNA sequencing

**DOI:** 10.18632/oncotarget.26361

**Published:** 2018-11-09

**Authors:** Lilla Ozgyin, Attila Horvath, Balint Laszlo Balint

**Affiliations:** ^1^ Department of Biochemistry and Molecular Biology, Genomic Medicine and Bioinformatic Core Facility, Faculty of Medicine, University of Debrecen, Debrecen H-4012, Hungary; ^2^ Department of Biochemistry and Molecular Biology, Nuclear Hormone Receptor Research Laboratory, Faculty of Medicine, University of Debrecen, Debrecen H-4012, Hungary

**This article has been corrected:** The correct affiliations information is given below:

^1^ Department of Biochemistry and Molecular Biology, Genomic Medicine and Bioinformatic Core Facility, Faculty of Medicine, University of Debrecen, Debrecen H-4012, Hungary

^2^ Department of Biochemistry and Molecular Biology, Nuclear Hormone Receptor Research Laboratory, Faculty of Medicine, University of Debrecen, Debrecen H-4012, Hungary

Original article: Oncotarget. 2018; 9:31312-31329. https://doi.org/10.18632/oncotarget.25764

