# Supplementary material for: Lyophilized human cells stored at room temperature preserve multiple RNA species at excellent quality for RNA sequencing
Source: Oncotarget. 2018 Jul 31;9(59):31312–29. doi: 10.18632/oncotarget.25764 (PMC6101130; doi:10.18632/oncotarget.25764)
Supplement: Supplementary file 1 [file oncotarget-09-31312-s001.pdf]

Lyophilized human cells stored at room temperature preserve multiple RNA species at excellent quality for RNA sequencing

SUPPLEMENTARY MATERIALS

Supplementary Table 1: Primer pairs used in the mRNA expression measurements in this study

Gene symbol	Forward primer (5′→3′)	Reverse primer (5′→3′)
GAPDH ('5'GAPDH')	GTGAACCATGAGAAGTATGACAAC	CATGAGTCCTTCCACGATACC
GAPDH ('3'GAPDH')	AGTCCCTGCCACACTCAG	ACTTTATTGATGGTACATGACAAGG
ACTB	CCCTGGCACCCAGCAC	GCCGATCCACACGGAGTAC
TCL1A	GGGAGGAATGGACAGACAGA	AGTGGGTGTGCAACATGAAA
RXRA	CCAGTACTGCCGCTACCAG	CATTCTCGTTCCGGTCCTT
TRERF1	AGGGTGAACCTCAGGAGACC	CAGGATTGCCAGTGACCAG
UBR2	CCTTCCTCTTTCCCTCCATC	CCAGGAGGCAGAGGTTGTAG
PTPRJ	GTCCTGTCCTAGGTGACATCG	GGAAGTCAGAAACTGGAACAGG
SLC6A4	CATTCTCGTTCCGGTCCTT	GTTGGCTATCGCTTCTGCAT

Supplementary Table 2: Primer pairs used in the eRNA expression measurements in this study

Assay name	Corresponding genomic region	Forward primer (5′→3′)	Reverse primer (5′→3′)
eIRF4_-1.9kb	chr6: 389707-389856	*TGGCAAATGAGTAAACCAGAAG*	TCAACATACCCTCCCCTCAC
eSPI1_-16kb	chr11: 47416340-47416446	CTCTGGGCAGGGTCACAG	*GGGCGCTTCCTGTTTTCT*
eMYC_-170kb	chr8: 128579211-128579280	*ACTCCAAAGTTCAAGCCCTCT*	GCACACCCGCTGTAACATT

Italicized oligonucleotides were used for gene-specific priming during the reverse transcription step. For reverse transcribing the normalizer, we used the *ACTB* reverse primer shown in Supplementary Table 1.

Supplementary Table 3: Primer pairs used in the lncRNA expression measurements in this study

Gene symbol	Forward primer (5′→3′)	Reverse primer (5′→3′)
MALAT1	AAAAAGCTACTAAAAGGACTGGTGTAA	*TCCAAATTCTTCTAACTCTTCCAAA*
GAS5	*GCCATGAGACTCCATCAGGC*	CCTCACCCAAGCTAGAGTGC
TUG1	CTGACGAAGACACCCATTCC	*GTGGAGGTAAAGGCCACATC*

Italicized oligonucleotides were used for gene-specific priming during the reverse transcription step. For reverse transcribing the normalizer, we used the *ACTB* reverse primer shown in Supplementary Table 1.

Supplementary Table 4: *P* values of the RT-qPCR data for selected mRNAs as calculated using paired *t*-test and related to Figure 2A

Gene symbol	GM12872	GM12873
UBR2	0.86	0.80
TRERF1	0.65	0.84
PTPRJ	0.39	0.78
SLC6A4	0.64	0.56
RXRA	0.58	0.22
TCL1A	0.54	0.68

Supplementary Table 5: Summary of basic RNA-Seq library information for each sample

Sample pair	Sample name	#raw reads	% deviation of UMRs from median	% deviation of DR from median	#expressed genes
**Pair 1**	**Control 1**	15 173 243	–2.6	+7.5	11 017
	**Lyo 1**	27 984 754	0.1	–12.9	10 918
**Pair 2**	**Control 2**	20 352 186	–0.1	+5.5	10 979
	**Lyo 2**	27 149 029	0.1	–6.8	10 881
**Pair 3**	**Control 3**	24 497 577	–0.2	+0.8	11 035
	**Lyo 3**	22 484 771	0.4	–0.8	10 970

UMRs: uniquely mapping reads; DR: duplication rate.

Supplementary Table 6: Differentially expressed genes with biotype annotations, FPKM values in the control and lyophilized cells and fold changes

Gene symbol	RNA biotype	FPKM (Control)	FPKM (Lyo)	Fold change (Control/ Lyo)
AGRN	protein coding	20.39	8.90	2.29
BICRA	protein coding	2.42	0.99	2.45
CIC	protein coding	8.90	4.26	2.09
FASN	protein coding	57.34	24.82	2.31
HCFC1	protein coding	15.14	7.40	2.05
INTS1	protein coding	14.64	7.55	1.94
KDM6B	protein coding	7.99	3.52	2.27
KMT2B	protein coding	7.71	3.61	2.14
KMT2D	protein coding	5.57	2.28	2.44
LENG8	protein coding	41.51	17.81	2.33
LINC01000	lncRNA	3.41	1.26	2.70
LINC01001	lncRNA	1.82	0.60	3.04
LINC01002	lncRNA	1.29	0.42	3.05
LINC01347	lncRNA	1.02	0.24	4.25
LOC100133331	lncRNA	2.54	0.86	2.95
LOC729737	lncRNA	2.30	0.70	3.30
MEGF8	protein coding	2.31	0.96	2.40
NCOR2	protein coding	21.84	9.77	2.24
NOTCH1	protein coding	6.83	3.08	2.22
PIEZO1	protein coding	24.40	11.15	2.19
PKD1P1	pseudogene	4.35	2.06	2.12
PLEC	protein coding	9.44	3.72	2.54
PLXNA3	protein coding	4.16	1.90	2.19
POLR2A	protein coding	30.26	12.81	2.36
PRR12	protein coding	4.91	1.92	2.56
PTPN23	protein coding	8.07	3.90	2.07
SETD1B	protein coding	2.52	1.10	2.29
TNRC18	protein coding	4.11	1.92	2.14

Supplementary Table 7: Transcript properties of differentially expressed genes

Gene symbol	cDNA length (bp)	cDNA GC%	CDS length (bp)	CDS GC%	5′ UTR length (bp)	5′ UTR GC%	3′ UTR length (bp)	3′ UTR GC%	ARE type
AGRN	7 394	67.5	5 793	68.5	1	100	1 137	61.7	-
BICRA	5 739	68.6	4 683	70.8	464	69.4	862	56.1	-
CIC	8 218	65.8	7 545	66.5	256	81.6	605	56.9	-
FASN	8 565	65.3	7 536	65.3	68	64.7	810	63.3	-
HCFC1	8 869	61.5	6 108	63.0	967	64.9	1 794	54.5	-
INTS1	6 959	65.2	6 573	64.7	68	73.5	300	70.7	intronic
KDM6B	6 713	62.4	5 049	64.2	N/A	N/A	1 275	55.0	3′UTR
KMT2B	8 469	63.5	8 148	63.7	77	77.9	321	58.6	-
KMT2D	19 419	64.3	16 614	60.1	389	63.2	2 805	52.9	-
LENG8	5 789	61.6	2 625	62.4	175	83.4	3 109	61.0	intronic
MEGF8	11 034	63.7	1 320	67.2	145	68.3	1 994	54.2	-
NCOR2	8 533	65.2	7 545	65.8	170	74.7	987	60.5	intronic
NOTCH1	9 306	62.9	7 668	65.8	N/A	N/A	1 638	49.5	-
PIEZO1	8 089	63.9	7 566	63.9	55	56.4	267	60.3	intronic, 3′UTR
PLEC	15 249	67.0	14 055	67.0	7.720	65.6	1 024	64.8	intronic
PLXNA3	10 885	57.4	5 616	62.3	12	66.7	5 094	51.0	3′UTR
POLR2A	2 083	55.8	1 701	56.4	86	82.6	237	43.9	-
PRR12	6 955	66.3	6 111	67.6	219	75.3	832	56.6	3′UTR
PTPN23	5 244	62.2	4 911	62.4	N/A	N/A	256	53.5	intronic
SETD1B	8 185	61.3	5 772	64.7	349	80.2	2 345	52.6	intronic, 3′UTR
TNRC18	10 572	67.1	8 907	68.4	194	70.6	1 316	55.0	intronic
LINC01000	10 263	56.3	N/A	N/A	N/A	N/A	N/A	N/A	N/A
LINC01001	5 007	61.7	N/A	N/A	N/A	N/A	N/A	N/A	N/A
LINC01002	4 874	65.3	N/A	N/A	N/A	N/A	N/A	N/A	N/A
LINC01347	3 834	52.7	N/A	N/A	N/A	N/A	N/A	N/A	N/A
LOC100133331	4 273	61.4	N/A	N/A	N/A	N/A	N/A	N/A	N/A
LOC729737	5 474	62.1	N/A	N/A	N/A	N/A	N/A	N/A	N/A

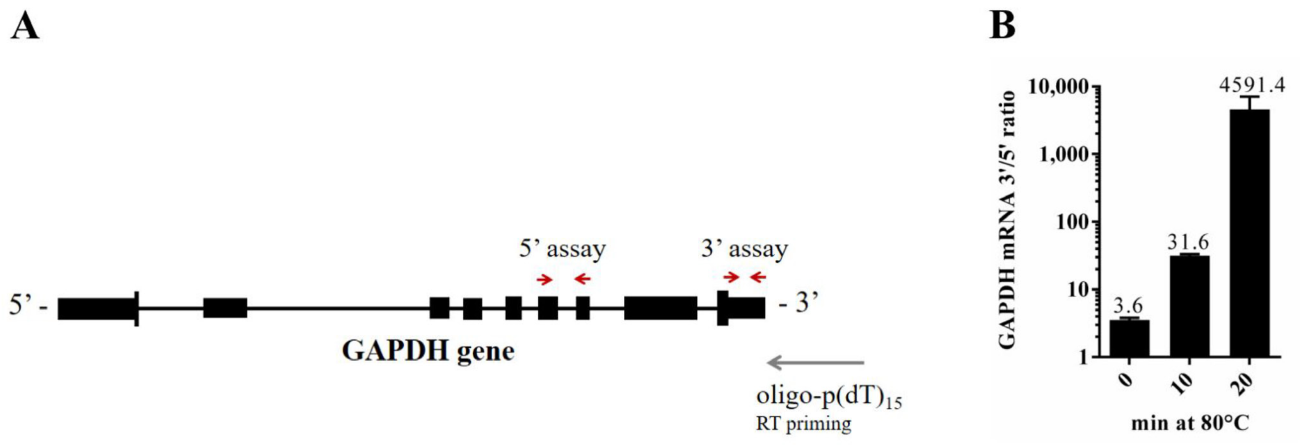

Supplementary Figure 1: Characteristics of the GAPDH mRNA 3′/5′ assay. (**A**) The location of RT and qPCR oligonucleotides on the GAPDH gene. (**B**) GAPDH 3′/5′ ratios measured for an intact sample (RIN = 10) and the same intact sample subjected to heat degradation for 10 and 20 min at 80° C (error bars represent the SD values of triplicate qPCR measurements).

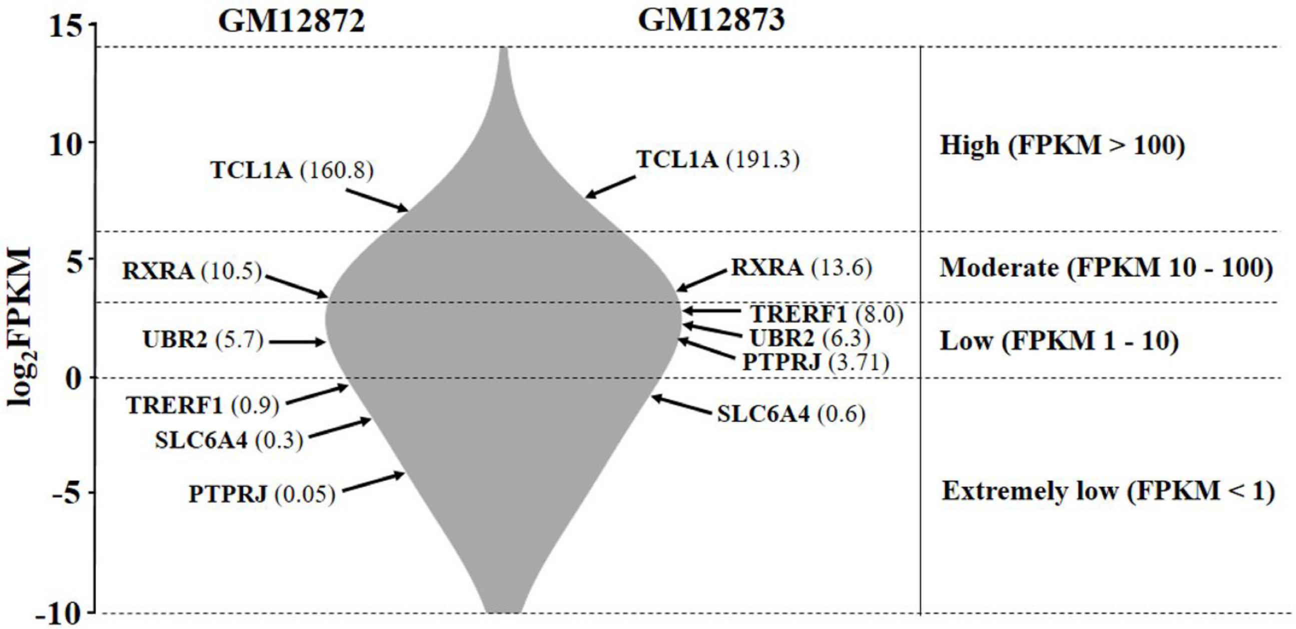

Supplementary Figure 2: FPKM distribution of genes based on RNA-Seq data from GM12873 used in selecting the target genes for validation. Selected genes are in bold, and their respective FPKM values in the given cell line are indicated in brackets.

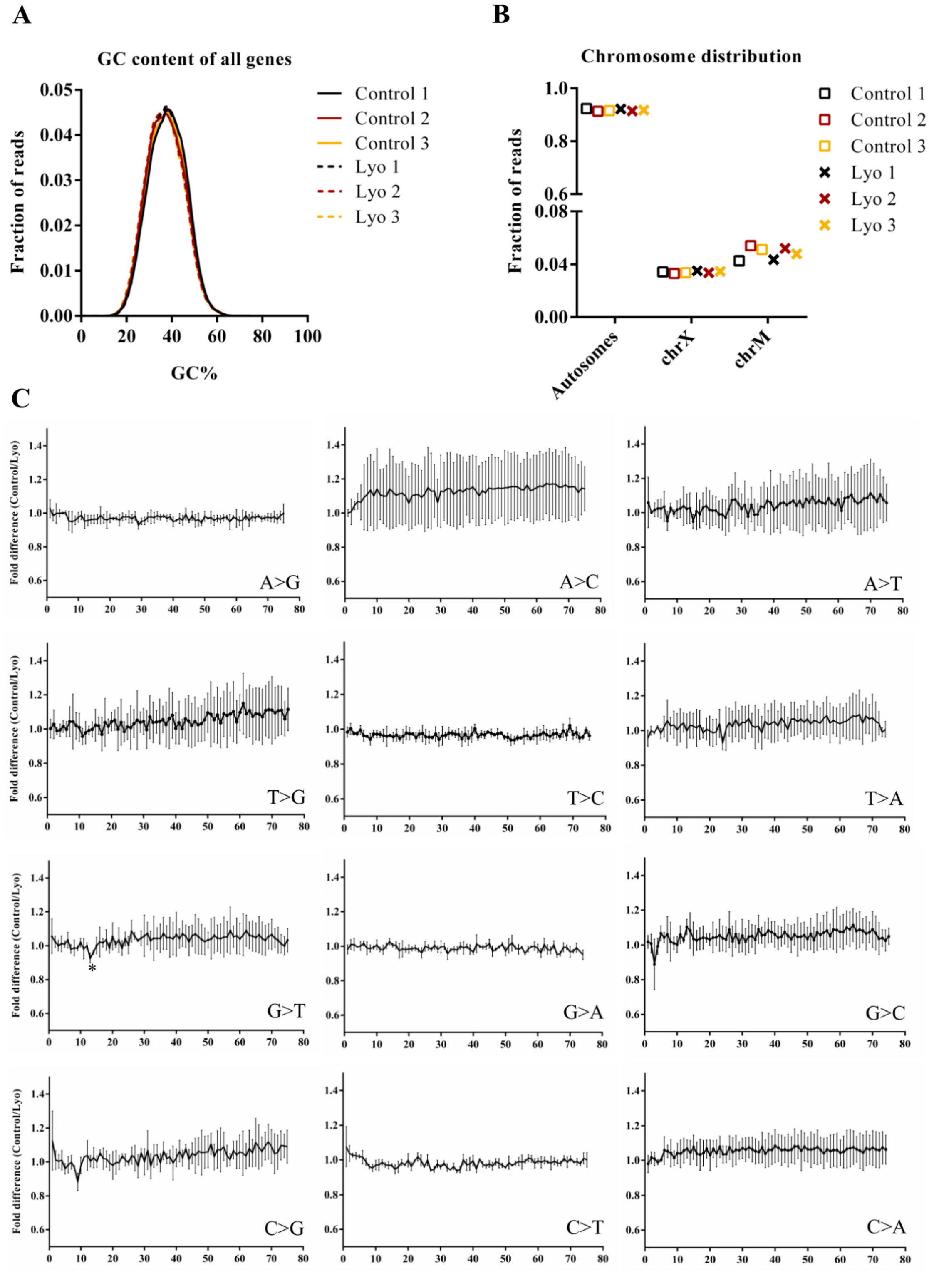

Supplementary Figure 3: Information of RNS-Seq reads related to GC content, chromosome distribution and mismatch ratios. (A) The fraction of reads possessing a particular GC content. After correction for multiple testing and at 5% FDR no significant difference was found between control and lyophilized samples (*P* values > 0.01 for all comparisons; *t*-test). (**B**) Chromosomal distribution of uniquely mapping reads (*P* values > 0.01 for all comparisons; paired *t*-test). (**C**) Per-base mismatch ratios (control/lyophilized) for all possible mismatch types. The lines connect the means of each group, while the error bars represent the SD. After correction for multiple testing and at 5% FDR no significant differences were found for any mismatch type at any read position (*P* values > 0.01; *t*-test) besides one position indicated with an asterisk (G>T, *P* = 0.004).

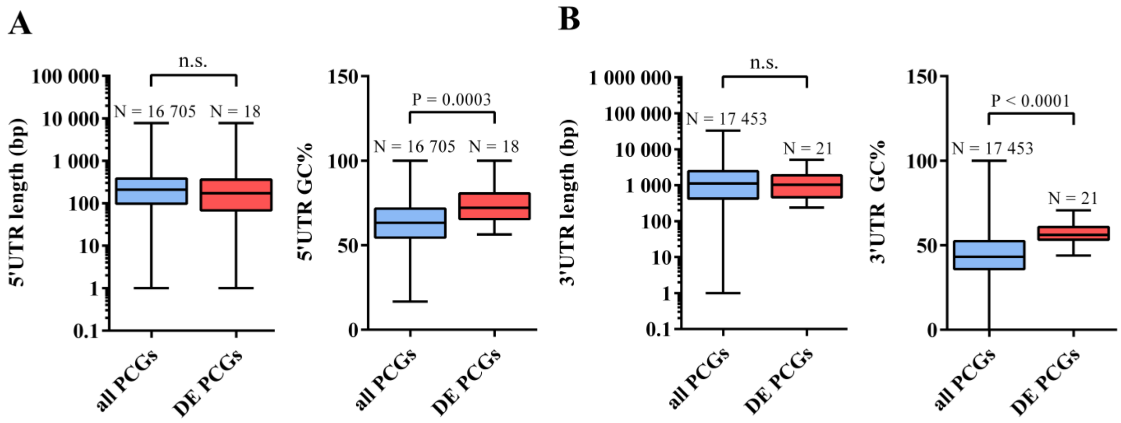

Supplementary Figure 4: The length and GC content of the UTRs of differentially expressed RNAs. **(A)** Box-and-whisker plots are showing the 5′ UTR lengths and GC counts of all human versus differentially expressed lncRNAs. (**B)** Box-and-whisker plots are showing the 3′ UTR lengths and GC counts of all human versus differentially expressed lncRNAs. All box-and-whisker plots display medians (horizontal line) and interquartile ranges (box) and minimum to maximum values (whiskers). Gene numbers and *P* values are indicated. DE = differentially expressed; PCG = protein-coding genes.
